# Hands on Methods for High Resolution Cryo-Electron Microscopy Structures of Heterogeneous Macromolecular Complexes

**DOI:** 10.3389/fmolb.2019.00033

**Published:** 2019-05-15

**Authors:** Marina Serna

**Affiliations:** Structural Biology Program, Spanish National Cancer Research Centre (CNIO), Madrid, Spain

**Keywords:** cryo-electron microscopy, single particle processing, macromolecular complexes, heterogeneity, refinement, resolution, psuedosymmetry

## Abstract

Electron microscopy of frozen hydrated samples (cryo-EM) is a powerful structural technique that allows the direct study of functional macromolecular complexes in an almost physiological environment. Protein macromolecular complexes are dynamic structures that usually hold together by an intricate network of protein-protein interactions that can be weak and transient. Moreover, a standard feature of many of these complexes is that they behave as nanomachines able to undergo functionally relevant conformational changes in one or several complex components. Among all the other main structural biology techniques, only cryo-EM has the potential of successfully dealing at the same time with both sample heterogeneity and inherent flexibility. The cryo-EM field is currently undergoing a revolution thanks to groundbreaking technical developments that have brought within our reach the possibility of solving the structure of biological complexes at atomic resolution. These technical developments have been mostly focused on new direct electron detector technology and improved sample preparation methods leading to better image quality. This fact has in turn required the development of new and better image processing algorithms to make the most of the higher quality data. The aim of this review is to provide a brief overview of some reported examples of single particle analysis strategies designed to find different conformational and compositional states within target macromolecular complex and specifically to deal with it to reach higher resolution information. Different image processing methodologies specifically aimed to symmetric or pseudo-symmetric protein complexes will also be discussed.

## Introduction

The complete understanding of how macromolecular complexes fulfill their intricate roles in the cell is the central theme in molecular biology. At the molecular level, precise knowledge of the structure of macromolecules in the cell critically contributes to the elucidation of their functional mechanism. The aim of structural biology is thus to, ideally, determine the 3D arrangement of the atoms of macromolecular complexes. Among the many different structural biology techniques, electron cryomicroscopy (cryo-EM) combined with single particle averaging techniques has recently emerged as a preferred option for solving near-atomic resolution structures of large macromolecular complexes and, is particularly effective in the case of heterogeneous samples, less amenable to other techniques (Bai et al., 2015a).

In cryo-EM flash frozen protein samples are irradiated with coherent electron beams and 2D projection images of the atomic density of many molecules in different orientations are recorded. Cryo-EM images of biological specimens typically display high noise levels due to the extremely low electron dose required to avoid radiation damage. To counter this effect and improve the signal to noise ratio it becomes necessary to align and average hundreds to thousands of single particle images corresponding to the same molecule orientation (Henderson and McMullan, 2013).

The last few years have seen the development of a new generation of direct electron detectors of peerless sensitivity and speed (McMullan et al., 2014, 2016), which coupled to other improvements in electron microscopes, allow for the acquisition of images of unprecedented high quality. Discovery and correction of beam-induced particle motion (Brilot et al., 2012; Campbell et al., 2012; Li et al., 2013; Scheres, 2014; Grant and Grigorieff, 2015; Zheng et al., 2017), automatic data acquisition routines, the development of new image processing strategies and faster computers (such as the use of GPUs) have made possible to attain near atomic-resolution cryo-EM maps with ease (Scheres, 2012; Yang et al., 2012; Elmlund et al., 2013; Grigorieff, 2016; Kimanius et al., 2016; Ludtke, 2016; Merk et al., 2016; Reboul et al., 2016).

Analysis of macromolecular complex structures solved during the so-called cryo-EM resolution revolution (Kühlbrandt, 2014) display atomic resolution details making it possible to elucidate important features that contribute to reveal their molecular mechanism. More importantly, those near-atomic resolution structures can be obtained from the most challenging specimens, including large macromolecular machines with dynamic composition and conformations (Nogales and Scheres, 2015; Fernandez-Leiro and Scheres, 2016).

## Heterogeneity Causes Resolution Anisotropy

Sample heterogeneity is an intrinsic feature of most macromolecular complexes as part of their dynamic mechanisms of action. Many cellular processes are driven by macromolecular complexes, which, far from being static, undergo functionally relevant conformational and compositional changes during their functional cycles. Thus, the control of this variability source in single particle image processing becomes of key importance to extract the maximum amount of useful information about the protein complex of interest.

In principle, structural variability poses a difficult challenge to overcome for 3D structure determination by cryo-EM, limiting severely the attainable resolution (Scheres, 2016). Regions of the structure that display heterogeneous features are incorrectly aligned and averaged with other images causing the loss of coherent signal. The ultimate consequence is that regions showing the highest heterogeneity become blurred or even invisible in the reconstruction, leading to less detailed and even incomplete maps.

**Figure 1:**
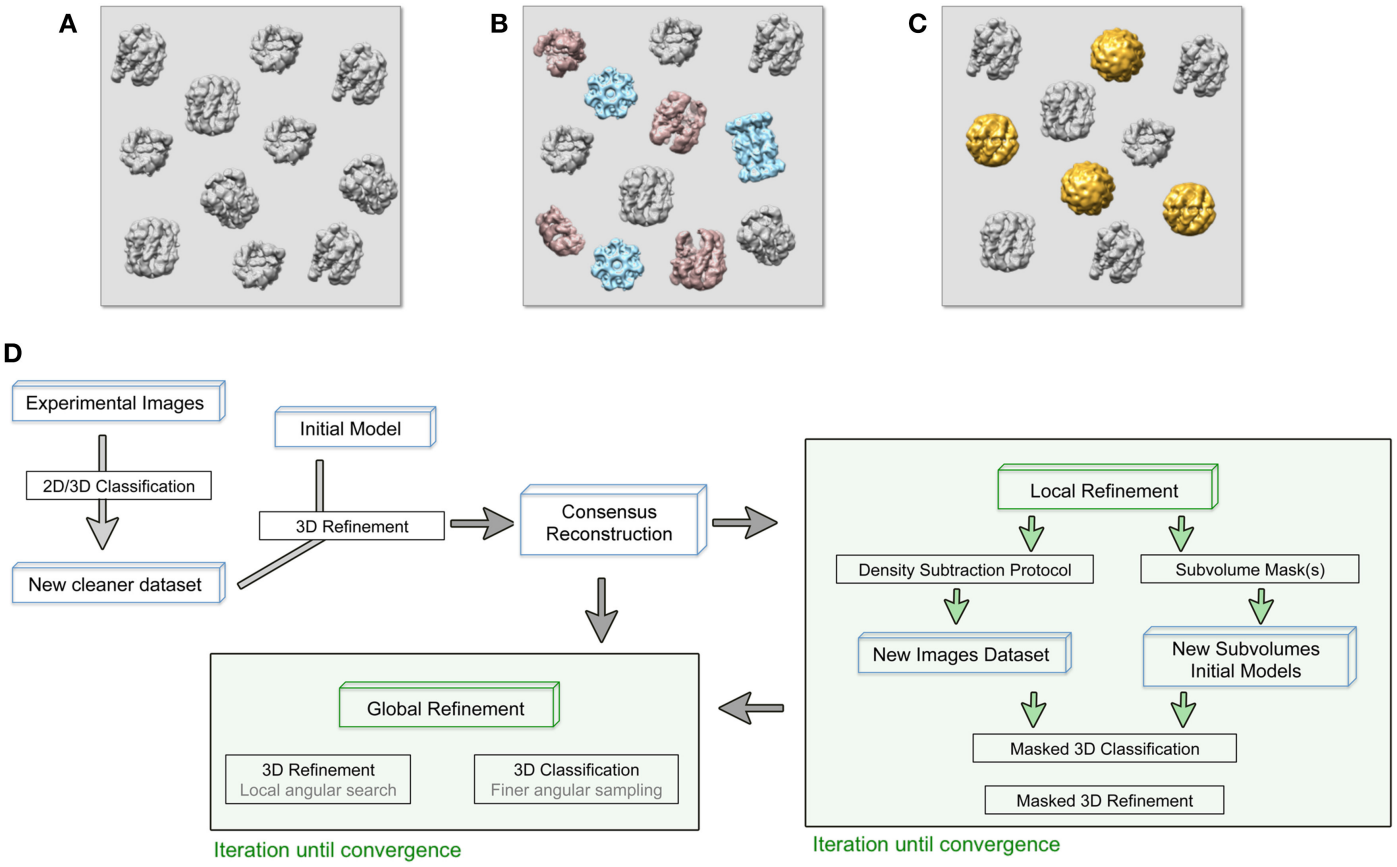
**(A–C)** Cryo-EM images are usually acquired on heterogeneous samples. Contaminants (light blue), denatured or partially disassemble complexes (light red) as well as different protein complex conformations (gold) are imaged together with the specimen (gray). **(D)** General approach workflow to handle sample heterogeneity during image processing. Processes (black boxes), iterative process (green boxes), and specific input data (blue boxes) are indicated. The consensus reconstruction is obtained from an initially heterogeneous dataset and it will be used as the initial reference for more specific refinement protocols to deal with sample heterogeneity.

There are several sources of sample heterogeneity that must be taken into consideration in order to deal with them (Figures 1A–C). Macromolecular assemblies are dynamic machines, which undergo conformational rearrangement in one or more protein subunits in order to perform their function (Alberts, 1998). This protein flexibility might be discrete or, most frequently, a continuum of different conformations of a particular protein subunit or multiple subunits moving independently from each other. The protein conformational landscape is also complicated by the inevitable co-existence in the sample of partially assembled complexes, partial subunit occupancy and/or even different protein subunit composition due to complex assembly dynamics (Figure 1). A particularly important case of protein complex heterogeneity is represented by symmetry mismatches.

Moreover, non-physiological heterogeneity may be introduced during sample and cryo-EM specimen preparation. On the one hand, most if not all samples cannot be purified to absolute homogeneity and contaminants are carried through the preparation process. On the other hand, cryo-EM sample preparation implies freezing the sample within a thin layer of amorphous water ice, introducing a stress factor that risks the occurrence of artifacts such as breakage of protein-protein interactions within the multi-subunit complexes, dissociation of the complex, and/or partial or complete denaturation caused by the collision of proteins with the extended air-water interface (Taylor and Glaeser, 2008). Last but not least important, are the errors introduced during image processing that will also affect the final reconstruction resolution. Among these, maybe the most significant will be bad particle selection due to limitations in the particle picking algorithms coupled to lack of user supervision (Henderson, 2013).

## Identification of Sample Heterogeneity

In cryo-EM, the resolution value provides a general criterion to evaluate the quality of solved macromolecular complexes structures and it is commonly estimated by the Fourier Shell Correlation method (Saxton and Baumeister, 1982; van Heel and Stöffler-Meilicke, 1985; Harauz and van Heel, 1986). Estimated resolution value also helps to define the structural details (e.g., secondary structure vs. aminoacid side chains) merited by a given reconstruction that can readily be interpreted and discussed. In this sense, the presence of sample heterogeneity is commonly manifested by impairment on achieving a high-resolution map. In general, global measurements of resolution do not report internal variation of map quality, as this resolution value will remain unaffected by small changes in the map. In this sense, a detailed inspection of the density map might reveal regions whose details agree with the global resolution value obtained while other regions are blurred. Those areas of lower resolution are usually caused by unaccounted sample heterogeneity. Thus, close examination of the local resolution variation has become critical to recognize and localize heterogeneity. Local resolution estimation was pioneered by ResMap (Kucukelbir et al., 2014), although currently most modern image processing packages include a dedicated local resolution routine [e.g., cisTEM Grant et al., 2018, cryoSPARC Punjani et al., 2017, RELION Scheres, 2012].

Upon detection of sample heterogeneity, this must in turn be characterized and, more importantly, classified in order to improve the complex reconstruction. In this sense, 2D and, in particular, 3D classification have been extensively employed to deal with partial occupancy and asymmetric reconstructions.

## 2D and 3D Classification to Identify and Classify Sample Heterogeneity

Sample heterogeneity can, in some cases, be recognized just by visual inspection of the obtained maps in the form of blurry regions in the density. However, for a detailed and more accurate analysis, dedicated software tools are necessary. Historically, many different approaches have been developed to perform the classification of heterogeneous cryo-EM data. Reference-based methods (Rossmann and Blow, 1962) were progressively substituted by statistical-based algorithms, including multivariate statistical analysis (MSA), and principal component analysis (PCA) (van Heel and Frank, 1981; Klaholz, 2015; Haselbach et al., 2017). Among the different 2D and 3D classification algorithms, those that rely on a regularized likelihood optimization algorithm like RELION have been proved to reliably deal with sample heterogeneity (Sigworth, 1998; Scheres et al., 2005; Scheres, 2012; Song et al., 2013; Chowdhury et al., 2015). Although successful, the maximum likelihood approach has some methodological limitations to be considered. On the one hand, these methods are prone to be trapped in local minima. This is particularly relevant when dealing with intrinsically noisy cryo-EM images and local optimization algorithms have been implemented in several image processing packages in order to overcome this problem (Sorzano et al., 2010; Elmlund et al., 2013; Punjani et al., 2017). In the particular case of macromolecular motions of continuous nature, discrete classes defined during ML-based image classifications will not address every conformation of the macromolecular complex. Alternative methods based on manifold embedding have also been proposed in order to map continuous heterogeneity in a data-specific coordinate system (Frank and Ourmazd, 2016).

For 2D classifications the RELION algorithm marginalizes only over in-plane orientations, which implies an inherent limitation in the capacity for structural sample heterogeneity identification. The user has to interpret the experimental projection images and this might lead to a miss-interpretation of the structural heterogeneity. Still this algorithm is very useful to identify bad particle alignment caused by protein partially unfolded, incorrectly selected or too closely selected particles.

On the other hand, the RELION 3D classification both contributes to the selection of a particle dataset suitable for high-resolution reconstruction and also to further identification of sample heterogeneity since the algorithm marginalizes over both the orientational and class assignments of the particles images (Scheres et al., 2007). Even if the number of classes is lower than the actual number of structures contained in the data set, an initial 3D classification would help to classify large differences in the structure. In order to find the proper number of classes for a given data set, as well as to assess reproducibility and consistency of the classification, it is recommended to test several rounds of classification using different initial references and different number of classes (Scheres, 2012). Subsequent 3D classifications with increasingly more exhaustive angular searches, as well as with the use of specific soft-edge mask, will shed light on smaller structural differences among the classes, contributing both to obtain a more homogeneous particle dataset for the high-resolution reconstruction and to identify sample heterogeneity (Scheres, 2016). FrealignX 3D classification included in the cisTEM software (Grant et al., 2018) represents a valuable alternative. Apart from using a ML approach (Lyumkis et al., 2013) it allows, as in RELION 3D classification, to restrict and focus the classification on a masked-in sub-volume. Another package, CryoSPARC (Punjani et al., 2017) includes a heterogeneous refinement routine that not only classifies but also refines heterogeneous 3D structures. Unlike RELION, CryoSPARC 3D classification and refinement requires a number of user-provided initial references to be refined simultaneously with the classified particle datasets. A more informative description of the conformational landscape of a macromocular complex can be obtained by a combination of 3D classification procedures with principal component analysis. An energy landscape is calculated where the conformational variations are plotted in a coordinate system (Clare et al., 2012; Haselbach et al., 2017).

## Focused Processing on Heterogeneous Structures

A general approach would take advantage of the 3D classifications to independently refine a particular class that could represent a homogeneous identity within the heterogeneous dataset (Figure 1D) (Bai et al., 2015b; Nguyen et al., 2016). Other, more focused approaches to treat heterogeneous sample are known as localized reconstruction methods and allow a more detailed analysis of small differences within the structures (Ilca et al., 2015; D'Imprima et al., 2017; Nakane et al., 2018).

As mentioned above, both masked refinement and masked classification are suitable tools to deal with the presence of multiple structures within the same data set. Both protocols start by applying a 3D mask to the reference at every iteration, including the density of interest while the rest of the complex is masked out. Thus, creating an adequate mask for the processing turns out to be essential. Moreover, validation of the reconstruction obtained should critically involve analysis of any masking effects.

Historically, masks have been applied during image processing to remove the solvent noise surrounding a molecular envelope, mainly at the alignment stage, and after 3D refinement when the gold-standard FSC calculation is used (Henderson et al., 2012; Scheres, 2012; Scheres and Chen, 2012). However, applying a mask is not only helpful to improve the resolution of the constant part of the complex but it is also the only effective way to isolate and extract heterogeneous regions to be treated as independent single particles for classification and high-resolution refinement purposes. This approach makes it important to closely test and monitor the created masks so they work as intended without introducing processing artifacts (Rawson et al., 2016).

Importantly, any generated masks should have soft edges rather than sharp edges in order to prevent artifacts in the Fourier domain during FSC estimations (Chen et al., 2013). Another important aspect to be considered in designing a mask for a 3D refinement is that including detailed features in the mask will lead to overfitting during the refinement, which causes an increase in the FSC curve that does not reflect the true signal-to-noise ratio and a consequent overestimation of the resolution (Rosenthal and Henderson, 2003; Chen et al., 2013). Thus, the reference volume that is used to create the mask is usually low-pass filtered.

## Focused Refinement Allows Reaching High Resolution Information in cryo-EM Reconstructions

Masked 3D refinement is a suitable tool to deal with datasets that contain flexible structures within the same protein complex. This method allows focusing the refinement protocol into a specific region within the subject structure (Louder et al., 2016; Nguyen et al., 2016). Through a masking operation that excludes everything but the region of interest through all the refinement iterations, only the target region signal is used for particle alignment and the map that comes out the refinement corresponds solely to the masked region (Nguyen et al., 2016; Nakane et al., 2018). The interface between the subvolumes might not be well-resolved, so it is important to analyse them in parallel with the unmasked refinement map of the entire volume (Scheres, 2016).

**Figure 2:**
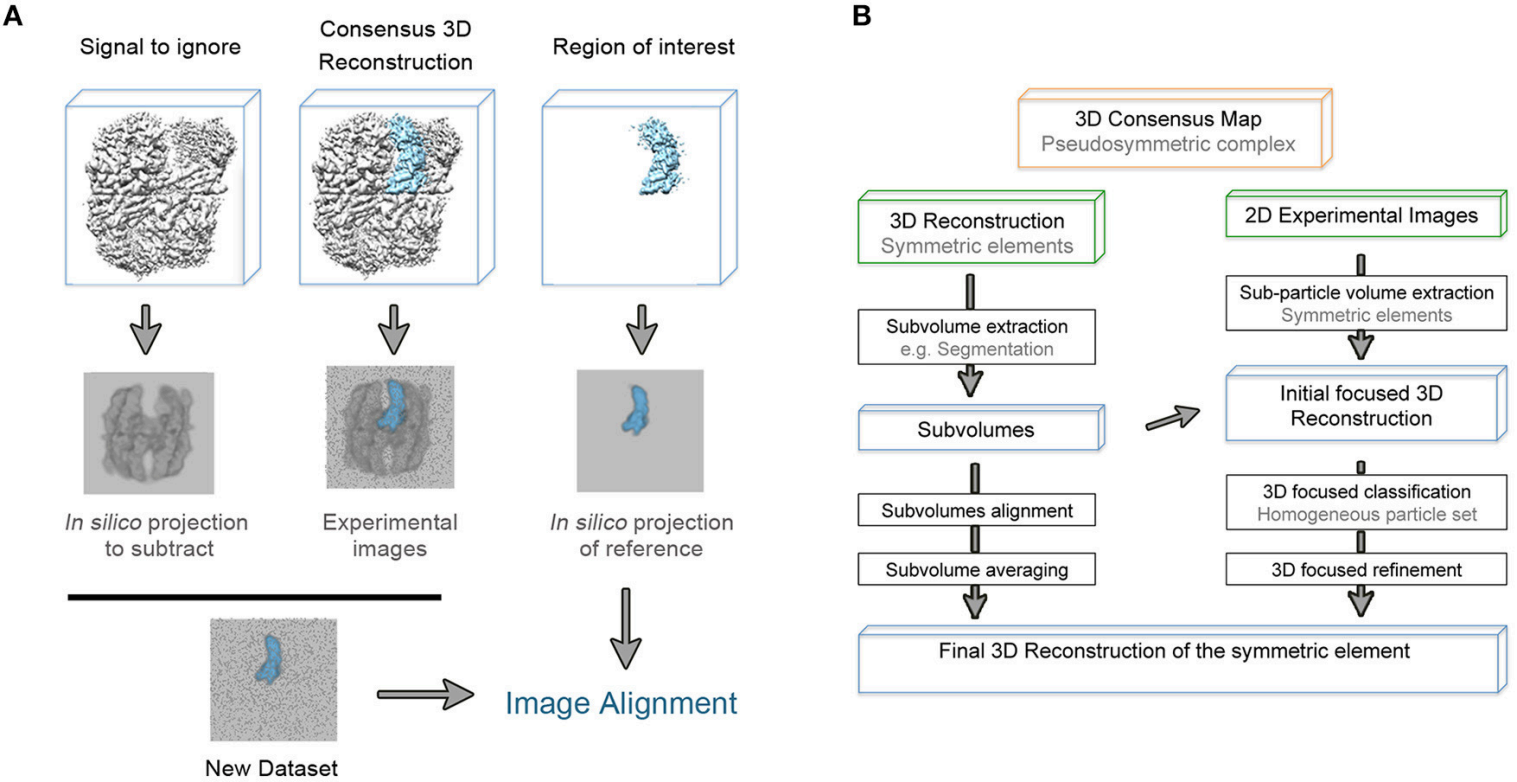
**(A)** Schematic representation of the density subtraction protocol. Generated mask from the initial map are apply to obtain both projections of the map region that will be subtracted from the experimental images and projections of the region that will be subjected to masked 3D classification and refinement [based on (Bai et al., 2015b)]. **(B)** General workflow to extract as much structural information from the pseudosymmetric features contained in a macromolecular complex.

It is worth mentioning that refinement in single particle analysis of cryo-EM data is understood as the refinement of particle orientations with respect to an initial reference. In the course of the masked 3D refinement, the designed mask is applied to the 3D structures so the given reference projections, that will be compared to the experimental images, only contain information about the region of interest. However, in this original approach, the subvolume mask is not applied to the experimental 2D images so they contain information about the entire particle. Thus, an inconsistency in comparing both reference and experimental projections occurs and the density of the experimental data that is not present in the reference projection acts as noise that might impaired orientational and class assignments (Bai et al., 2015b; Ilca et al., 2015). In order to overcome this problem, and based on previous structural studies of symmetry mismatches in bacteriophage phi29 (Morais et al., 2003) and flaviviruses (Zhang et al., 2007), a density subtraction protocol was developed (Figure 2A). Subtraction of the experimental signal that is masked out in the 3D reference is carried out at the 2D images level so that the comparison inconsistency mentioned is minimized. It is highly recommended to validate that the density was correctly subtracted by calculating a 3D reconstruction of the subtracted particles. Finally, the new subtracted experimental images dataset can be used both for masked 3D refinement and classification where some parameters such as orientational searches can be fine-tuned to improve particle alignment (Nguyen et al., 2015; Scheres, 2016). Very recently, a new method based on the signal-subtraction and masked 3D refinement protocol has been specifically designed for simultaneous refinement of a number of regions of interest within the same protein complex.

Another consideration is that macromolecular complexes can often display continuous motions rather than discrete conformational stages, and the understanding of those motions might be critical to functionally characterize the protein complex. Normal mode analysis has been used to deduce macromolecular motions for low-resolution maps (Tama et al., 2002) and the previously discussed masked 3D classification and refinement protocols allow reconstruction of the bodies that have relative motions within the macromolecular complex. Recent contributions in the field allow for the reconstruction of much smaller regions of a given map (Schilbach et al., 2017). Based on this, a new method called multibody refinement, uses the entire dataset in the iterative 3D refinement of previously defined individual bodies (Nguyen et al., 2015; Nakane et al., 2018). Moreover, it also provides a principal components analysis to monitor relative motions between the reconstructed bodies. CryoSPARC's non-uniform refinement is also aimed to improve resolution of disordered or flexible regions by reducing over-fitting tendency of disordered regions (Punjani et al., 2017).

## Pseudo-symmetry in Macromolecular Complexes as an Advantageous Feature for Single Particle Refinement

A fair number of macromolecular complexes present some kind of internal symmetry or pseudo-symmetry, when more than one copy of the same structural element is not related by the normal symmetry of the complex. Symmetry is both a relevant feature in the biological function of the complex and a very useful feature that can be taken advantage of in single particle image processing. The application of symmetry during the 3D refinement results in practice in the multiplication of the number of useful particles or the structural elements that are symmetrically related.

The signal might be boosted through the application of symmetry in the low-resolution range, while it would impair protein alignment and impede attaining high-resolution when incorrect symmetry operators are imposed. Some complexes can be considered symmetric at low resolution with asymmetric features becoming observable only at high-resolution. Therefore, it is crucial to determine and demonstrate the nature of the macromolecular complex symmetry so the final volume corresponds to the actual protein structure.

Beyond the mere examination of a model that has more than one copy of a chain, several methods to identify internal symmetry and pseudo-symmetry in a structure have been developed. In particular, Phenix has implemented a tool based on direct search for patterns of density that are present in more than one place in the map (Terwilliger, 2013). The software refines the correlation values between the identified symmetric element and the rest of the map so final orientations and translations that defined the pseudo-symmetry are provided. Once the pseudo-symmetry is defined, and based on its nature, there are different approaches to apply it during structure refinement (Figure 2B).

## Classical Approach to Pseudo-symmetric Maps

Prior to the development of the 3D masked classification and refinement to improve resolution for pseudo-symmetric complexes, parallel refinements were usually carried out with and without symmetry application. The aim was to impose symmetry during the refinement of the molecular complex, enforcing signal for its symmetric part. The density map improvement would be obtained at the cost of blurring the asymmetric regions. In the end a chimeric map would be built using the symmetric density map obtained from the 3D refinement where the symmetry was imposed, and the asymmetric density map where the symmetry was not considered. Several macromolecular complex structures have been solved thanks to this approach (Coloma et al., 2009).

An evident issue of the method described appears at the interface between both independently refined maps where the information between the connected areas will be lost. Moreover, due to the misalignment of the asymmetric part during the symmetry-imposed 3D refinement, the achieved resolution will be necessarily limited. Over time, new methods have been developed to overcome these limitations.

## Density Maps of Identical Regions of a Macromolecular Complex can be Aligned and Average to Boost Structure Resolution

When a pseudo-symmetric protein complex contains more than one copy of the same object, it is possible to manually apply the symmetry directly to the symmetric area of the 3D reconstruction. The reasoning behind this method is the same as in the subtomogram averaging approach (Wan and Briggs, 2016). Subvolumes of each symmetric element of the macromolecular complex could be extracted from the overall structure map, aligned and averaged. Density signal will be then boosted and higher resolution maps obtained. Tools for subvolume extraction, maps alignment and averaging can be found in many image processing packages, as well as in 3D structures visualization software such as Chimera (Pettersen et al., 2004).

The potential of this method is greater when the alignment and average of the identical regions of the macromolecular complex can be performed at the level of 2D experimental images rather than using 3D reconstructed volumes. This idea has allowed the development of new powerful methods such as the symmetry-expansion approach (Ilca et al., 2015; Kimanius et al., 2016; Zivanov et al., 2018).

## Symmetry-expansion Approach

In a symmetric molecular complex, individual and symmetric elements of the structure can be conformationally heterogeneous, making the complex asymmetric. In this case, each density element is subtracted from the overall structure and treated as single particles that are related by the overall symmetry of the complex. The expanded dataset is obtained by applying to the 2D experimental images the corresponding transformation calculated from the known overall symmetry of the complex coupled with the subtraction operation. The structure of the asymmetric feature is then obtained through a combination of 3D masked classification and refinement strictly with local angular searches (Zhou et al., 2015).

This approach was originally applied by Briggs et al. (2005) to refine the vertices of the Kelp fly virus capsids and was initially implemented in RELION for cyclic symmetries (Kimanius et al., 2016). More recently, a new version of this method allows dealing also with molecular complexes whose asymmetrical feature is not related to the overall symmetry of the complex (Zivanov et al., 2018). Given a density map, this local symmetry method creates one mask per identical region and defines a unique operator that allows rotation of a masked region to match with another identical region that are symmetry-related. Initial operators are estimated from global orientation searches on the three euler angles and then, they are refined using progressively finer angular and translational searches. Final optimized operators are combined to apply the local symmetry to the subsequent 3D classification and reconstruction. This method can be also iteratively applied to improve the final structure.

## Conclusions

Since the earliest applications of the technique, the compositional and conformational dynamics of protein complexes have represented a challenge for EM image processing and single particle analysis. Nowadays this paradigm has begun to change thanks to the development of a new generation of computational tools that take full advantage of the recent revolutionary advances in EM hardware and data acquisition protocols. As for what lies ahead for the field, there is still much room for further improvements on current strategies and the development of wholly new strategies for image data analysis.

## Author Contributions

The author confirms being the sole contributor of this work and has approved it for publication.

### Conflict of Interest Statement

The author declares that the research was conducted in the absence of any commercial or financial relationships that could be construed as a potential conflict of interest.
